# Prediction for optimal dosage of pazopanib under various clinical situations using physiologically based pharmacokinetic modeling

**DOI:** 10.3389/fphar.2022.963311

**Published:** 2022-09-12

**Authors:** Chunnuan Wu, Bole Li, Shuai Meng, Linghui Qie, Jie Zhang, Guopeng Wang, Cong Cong Ren

**Affiliations:** ^1^ Department of pharmacy, Key Laboratory of Cancer Prevention and Therapy, Tianjin Medical University Cancer Institute and Hospital, Tianjin, China; ^2^ National Clinical Research Center for Cancer, Tianjin, China; ^3^ Tianjin’s Clinical Research Center for Cancer, Tianjin, China; ^4^ Zhongcai Health Biological Technology Development Co., Ltd., Beijing, China; ^5^ Department of pharmacy, Liaocheng People’s Hospital, Liaocheng, China

**Keywords:** pazopanib, PBPK model, dosing regimen adjustment, hepatic impairment model, food effect

## Abstract

This study aimed to apply a physiologically based pharmacokinetic (PBPK) model to predict optimal dosing regimens of pazopanib (PAZ) for safe and effective administration when co-administered with CYP3A4 inhibitors, acid-reducing agents, food, and administered in patients with hepatic impairment. Here, we have successfully developed the population PBPK model and the predicted PK variables by this model matched well with the clinically observed data. Most ratios of prediction to observation were between 0.5 and 2.0. Suitable dosage modifications of PAZ have been identified using the PBPK simulations in various situations, i.e., 200 mg once daily (OD) or 100 mg twice daily (BID) when co-administered with the two CYP3A4 inhibitors, 200 mg BID when simultaneously administered with food or 800 mg OD when avoiding food uptake simultaneously. Additionally, the PBPK model also suggested that dosing does not need to be adjusted when co-administered with esomeprazole and administration in patients with wild hepatic impairment. Furthermore, the PBPK model also suggested that PAZ is not recommended to be administered in patients with severe hepatic impairment. In summary, the present PBPK model can determine the optimal dosing adjustment recommendations in multiple clinical uses, which cannot be achieved by only focusing on AUC linear change of PK.

## 1 Introduction

PAZ is a multi-targeted kinase inhibitor that primarily targets the vascular endothelial growth factor receptor (VEGFR), and is clinically indicated for the treatment of patients with advanced renal cell carcinoma (RCC) (Schutz et al., 2011; Food and Drug Administration, 2021). PAZ, developed by Novartis, is the third tyrosine kinase inhibitor and the sixth targeted agent approved by FDA for treatment of RCC, and received initial approval in 2009 (Keisner and Shah, 2011). Dosage strengths were approved as 200 mg tablets for oral administration of 800 mg OD for patients with normal hepatic function (Food and Drug Administration, 2021).

PAZ is a weak base compound (pKa of 2.1, 6.4) showing pH-dependent with very slight solubility at pH = 1.0 and being practically insoluble at above pH 4.0 (Herbrink et al., 2018). PAZ is considered a highly permeable compound (Fink, 2020) and is classified as a class II drug under the Biopharmaceutics Classification System (BCS) (Evaluation, 1997). Accordingly, the rate and extent of *in vivo* PAZ solubility can appreciably affect the peak time and amount of absorbed drug. The mean oral bioavailability of PAZ after an oral 800 mg dose in humans is only approximately 21.4% (Deng et al., 2013) because of very incomplete absorption caused by poor solubility. Furthermore, as the oral dose is reduced, PAZ bioavailability increases significantly, with a 400 mg dose having a 40% higher bioavailability than an 800 mg dose (Food and Drug Administration, 2009). Lower gastric solubility at higher doses is likely to result in a huge difference in PAZ available for absorption between different doses.

PAZ undergoes moderate metabolism by CYP3A4 enzyme, with making minor metabolism contributions by CYP1A2 and CYP2C8 enzyme (Food and Drug Administration, 2009; Boudou-Rouquette et al., 2016). The primary metabolic pathways of PAZ were identified to be mono-and di-oxygenation metabolism in the liver (Food and Drug Administration, 2009). It was reported that at least seven metabolites were present in human plasma, urine, and feces (Food and Drug Administration, 2009). Of these metabolites, only metabolite GSK1268997 exhibits equal potency towards VEGFR as PAZ, and the other metabolites against VEGFR are less potent in comparison to PAZ (Heath et al., 2010). Whereas, systemic exposure of GSK1268997 is only about 2% of unchanged PAZ (Heath et al., 2010), and the metabolite GSK1268992 with the highest concentration in humans only accounts for approximately 6% of the total dose (Food and Drug Administration, 2009). Furthermore, *in vitro* data indicated that PAZ was a weak substrate for the efflux transporters P-gp and a moderate substrate for BCRP1 (Minocha et al., 2012; Boudou-Rouquette et al., 2016). Similarly, a recent study found that the transporter OCT1 plays a major role in the hepatic uptake of PAZ (Ellawatty et al., 2018). Moreover, PAZ is also a weak inhibitor of CYP3A4 and CYP3D6, and similarly, it is also a moderate inducer of CYP3A4 and CYP2B6 (Food and Drug Administration, 2009; Yamada et al., 2020). Similarly, *in vitro* studies (Xu et al., 2010) revealed that PAZ inhibited the UGT1A1 metabolizing enzyme with an IC_50_ of 1.2 μM and the OATP1B1 transporter with an IC_50_ of 0.79 μM. Besides, a previous *in vitro* study reported the inhibition of PAZ on three kidney transporters, with a K_i_ of 3.0 μM at OCT2, of 1.7 μM at MATE1 and of 3.3 μM at MATE2-K, respectively (Sauzay et al., 2016). PAZ is primarily eliminated via feces with approximately 82.2% administered radioactivity dose and 67% the unchanged drug (Deng et al., 2013), and undergoes minor renal elimination with approximately 2.6% of the administered dose (Deng et al., 2013).

A large number of factors can affect human PK variables (i.e., AUC: area under plasma concentration vs time curve, C_max_: peak concentration, C_trough_: trough concentration at steady state, and T_max_: time to peak) of PAZ, such as co-administration with CYP3A4 modulators (drug-drug interaction, DDI), in combination with drugs elevating gastric pH (acid-reducing agents), food effects, insufficient hepatic function, and genetic polymorphisms of CYP3A4. Previous research found that co-administration with ketoconazole (a strong CYP3A4 inhibitor) resulted in an approximately 66% increase in AUC of PAZ, and a 30% decrease when combined with carbamazepine (Van Leeuwen et al., 2014) (a moderate CYP3A4 inducer). In addition, with lapatinib (moderate competitive CYP3A4, P-gp and BRCP inhibitor), the AUC of PAZ was increased by 59% (Van Leeuwen et al., 2014). Similarly, concomitant use with drugs that increase gastric pH reduced PAZ oral bioavailability by approximately 39% (Budha et al., 2012) and significantly decreased clinicalo efficacy (Mir et al., 2019). Additionally, the effects of food and hepatic impairment on the PK variables (AUC, C_max_ and C_trough_) of PAZ have been investigated (Heath et al., 2010; Shibata et al., 2013). In addition, exposure-response relationships in patients have also been deeply investigated in clinical trials. In the use of PAZ, multiple studies suggested a minimum concentration (C_trough_) of ≥20 μg/ml as a PK threshold for optimal clinical efficacy (Verheijen et al., 2017). On the other hand, exposure-toxicity analyses have been carried out. It was clinically observed that increased blood pressure in patients was strongly associated with a plasma concentration of PAZ (Food and Drug Administration, 2009; Verheijen et al., 2017). When C_trough_ is above 32 μg/ml in a clinical setting, the incidence of high blood pressure can reach nearly 80% (Food and Drug Administration, 2009). Moreover, a strong correlation between hepatotoxicity and PAZ concentration was also observed. ALT and AST, as surrogates of liver toxicity, elevation have been observed with higher PAZ C_trough_. When C_trough_ is above approximately 56 μg/ml, probability of ALT elevation approaches 20% (Food and Drug Administration, 2009). A recent study suggested that the mechanism of hepatotoxicity was the inability to metabolize bilirubin in certain patients with UGT1A1 genetic variants (such as UGT1A1*28) (Henriksen et al., 2020). Similarly, the latest investigation reported another underlying mechanism in which reactive metabolites of PAZ with aldehyde structures were involved in liver injury (Paludetto et al., 2020). However, regardless of which toxicity mechanism, C_trough_ plays a key part in hepatotoxicity.

Currently, three papers are involved in the development of PBPK model of PAZ (Fink et al., 2020a; Fink et al., 2020b; Riedmaier et al., 2020). Nevertheless, two papers among them aimed to evaluate the key role of low solubility in drug absorption (Fink et al., 2020a; Fink et al., 2020b). The work by Riedmairer primarily focused on the influence of food on multiple drug absorptions (Riedmaier et al., 2020). However, in the developed PBPK model for PAZ (Riedmaier et al., 2020), there was an inability to evaluate the effect of food on PAZ oral absorption.

When taking PAZ in patients, systematic exposure, clinical efficacy, and safety should be considered fully and be then determined for the optimum dosing regimens in various clinical situations, such as DDI influence, with food and administration in patients with liver dysfunction, etc. Therefore, we developed a PBPK model and used this model to 1) simulate the PK profiles of PAZ in patients in various different dosage regimens, respectively; 2) simulate the PK alterations of PAZ when concomitant use with CYP3A4 inhibitors, with esomeprazole, with food, and in patients with liver dysfunction; and 3) recommend an acceptable dosing regimen in the general patient population, given the strong efficacy and mild safety profile.

## 2 Materials and methods

### 2.1 Materials

PAZ drug substance, a purity of 98.0%, was purchased from Shanghai Titan Scientific Co., Ltd. FaSSGF, FEDGAS (pH3, 4.5, 6), FaSSIF v2 and FeSSIF v2 were purchased from Biorelevant Ltd. (Britain). Bile salt (Sodium taurocholate), a purity of 97%, was purchased from Shanghai yuanye Bio-Technology Co., Ltd. Ammonium acetate (analytical grade) was purchased from Beijing Kulaibo Technology Co., Ltd. Maleic acid (analytical grade) was purchased from Beijing Huawei Ruike Chemical Co., Ltd. Vandetanib, a purity of 98%, was purchased from Beijing Jingming Biotechnology Co., Ltd. An individual human cDNA-expressed CYP3A4 recombinant enzyme was purchased from Cypex Ltd. (Britain). NADPH regenerating system (containing 1.3 mmol/L NADP^+^, 3.3 mmol/L glucose 6-phosphate, 3.3 mmol/L MgCl2, and 0.4 U/mL glucose-6-phosphate dehydrogenase, 0.05 mmol/L sodium citrate) was purchased from Beijing Huizhi Heyuan Biotechnology Co., Ltd. The Tris buffer was ultra-pure grade and purchased from Beijing Solarbio Science &Technology Co., Ltd. Acetonitrile with chromatographic purity was purchased from Thermo Fisher Technology Co., Ltd. Hydrochloric acid solution was analytical grade and purchased from Beijing Tongguang Fine Chemical Company. Phosphoric acid with chromatographic purity was purchased from Tianjin Guangfu Fine Chemical Research Institute. Dimethyl sulfoxide was not less than 99% and purchased from Tianjin Balance Bio-tech Co.,Ltd. Sodium hydroxide and potassium dihydrogen phosphate were analytical grade and purchased from Beijing Chemical Works.

### 2.2 *In vitro* solubility testing

In this work, the equilibrium solubility measurement method was used to determine the solubility of PAZ in multiple conditions. Besides, we also determined the effect of concentration of bile salt (sodium taurocholate, NaTC) on the increase in solubility of PAZ in this study. Solubility measurements were conducted in a centrifuge tube at 37°C for 2 h in a shaking water bath, in which multiple biorelevant media and excess PAZ substance were contained. After incubation of 2 h, 1 ml aliquot of each sample was withdrawn from the tube. The samples were filtered using a 0.22 μm filter, the first 0.8 ml was discarded, and the remaining 0.2 ml was analyzed by the HPLC. The specific experiments containing different conditions are as follows:

i) Solubility testing in simulated fasted and fed media

The purpose of this experiment is to determine solubility data of PAZ in simulated human gastrointestinal tracts and then load that data into PBPK model for a more accurate prediction of PK in fasted and fed states. Specifically, solubility media were prepared using different phosphate buffers. Media pH was adjusted to the required value (Table 1, target value) using KOH or H_3_PO_4_, which were equal to the pH value of gastrointestinal tract compartment in a fasted and fed state in humans, respectively.

ii) Biorelevant solubility

**TABLE 1:** pH-solubility at fasted/fed state.

Test medium	Solubility (mean ± SD, μg/mL) at fasted state	Test medium	Solubility (mean ± SD, μg/mL) at fed state
FaSSGF-PH1.3^a^	436.1 ± 1.0	FEDGAS-PH3.0^b^	322.7 ± 2.6
PH2.0	263.9 ± 1.1	FEDGAS-PH4.5^b^	147.0 ± 1.9
PH3.0	391.4 ± 4.6	FEDGAS-PH4.9^b^	40.0 ± 0.7
PH4.5	68.3 ± 3.3	FeSSIF-V2-PH5.8^b^	6.5 ± 1.3
PH5.0	24.2 ± 2.3	PH7.0	N
PH6.0	7.60 ± 0.6	PH7.5	N
FaSSIF-V2-PH6.5^b^	0.63 ± 0.18	—	—
PH7.0	N	—	—
PH7.5	N	—	—
FaSSGF-PH4.78 ^b^	46.1 ± 3.4	—	—

N: Not detected (below the detection limit); -: no data; SD: standard deviation.

a The pH of FaSSGF, was adjusted to 1.3 to simulate gastric pH.

b Biorelevant medium (see “2.2 *In vitro* solubility testing” section for more details).

This experiment aims to determine PAZ solubility in a variety of biorelevant medium, including FaSSGF (simulated gastric fluid at fasted state), FeSSGF (simulated gastric fluid at fed state), FaSSIF-v2 (simulated intestinal fluid at fasted state), FeSSIF-v2 (simulated intestinal fluid at fed state), and FEGGAS (pH 3.0, early state after high-fat meal), FEGGAS (pH 4.5 middle state after high-fat meal), FEGGAS (pH 4.9 late state after high-fat meal). The every medium (10 ml) was separately added to the tubs along with excess PAZ for solubility measurement in biorelevant media.

Additionally, the pH of FaSSGF was adjusted to 4.78 with KOH solution, followed by addition of excess PAZ, to determine solubility. The purpose of this experiment is to evaluate solubility change under the condition of elevated gastric pH after co-administration of PAZ with esomeprazole.

iii) NaTC effect

This experiment aims to evaluate the influence of NaTC concentration on solubility of PAZ, and then to introduce these data into a hepatic impairment simulation model for an estimate of PK in patients with liver dysfunction. Specifically, PAZ solubility was investigated at seven different NaTC concentrations (0, 0.937, 1.87, 3.75, 7.5, 15, and 30 mM (Mithani et al., 1996)) to estimate enhancement of NaTC on solubility of PAZ. In this experiment, a final pH of 5.5 was used, representing a mean front luminal pH at fed state.

### 2.3 *In vitro* metabolism assay

In this study, the substrate depletion method was used to investigate PAZ metabolism by the P450 enzyme. Metabolism experiments were performed in 100 nM Tris-HCl buffer (pH7.4) with PAZ (1.0 μM) and human recombinant CYP3A4 enzyme (5 nM). After preincubation at 37°C for 5 min in a shaking water bath, and the reaction was initiated by the addition of 60 μl NADPH regeneration system in a final volume of 1.0 ml. PAZ stock solution was prepared with DMSO, and the final concentration of DMSO was 0.1% of the total volume. Aliquots (100 μl) of the reaction mixture were removed at time points of 0, 10, 20, 30, 45, 60, and 90 min, followed by the immediate addition of an equal volume of ice-cold acetonitrile containing 1 μg/ml vandetanib as an internal standard to quench the reaction. The mixture was mixed for 2 min, cooled for 5 min on ice, and then centrifuged at 10,000 rpm for 10 min at 4°C. The supernatant was analyzed by high performance liquid chromatography (HPLC). The reactions were carried out in triplicate, and in the meantime, a negative control without the addition of NADPH regeneration system was performed. Experiment data were given as the mean and standard deviation (SD) from three independent experiments. Intrinsic clearance of PAZ by CYP3A4 (CYP3A4CL_int_) was calculated as follows:

Remaining PAZ (%)=Exp(−k×t)∗100 (1)

$$CYP3A4{CL}_{int}=k\times V/C_{enzyme}$$ (2)

Where k is rate constant (min^−1^); t is incubation time (min); V is final reaction volume (1.0 ml); C_enzyme_ is CYP3A4 concentration (5 pmol).

### 2.4 HPLC method

The chromatographic separation was carried out using a HPLC system with an X-Bridge C_18_ column (250 mm × 4.6 mm, 5 μm, Waters) and at a column temperature of 30°C. The mobile phase consists of solvent A (water containing 20 mM ammonium acetate) and solvent B (acetonitrile) (60:40). The flow rate was set at 1.0 ml/min for a total run time of 10 min. The retention time of PAZ is 7.3 min under this chromatographic condition, and PAZ is determined by an internal standard method (vandetanib as an internal standard).

### 2.5 PBPK model development and verification

#### 2.5.1 PBPK model development

The PK-Sim^®^ (Version 10.0, Bayer Technology Services, Leverkusen, Germany) was utilized to establish the PBPK model; Digit (Version 1.0.4, Simulations Plus, United States ) was used to digitize the figures of PK profiles of PAZ in humans.

When developing the PBPK model of PAZ, two key parameters were needed, i.e., the pH-solubility data in multiple different biorelevant media and CYP3A4CL_int_. Whereas, these two parameters cannot be obtained from published literatures. Hence, the two parameters were determined using the above vitro experiments (see above 2.2 and 2.3). In order to better describe the tissue distribution of PAZ, Rodgers and Rowland and PK-Sim standard methods were used to estimate tissue distribution and cellular permeability, respectively. The clearance of PAZ in this PBPK model was estimated with a combination of CYP3A4 metabolism, hepatic clearance (CL_A_) and renal clearance (CL_R_). The CYP3A4 metabolism was calculated using CYP3A4CL_int_, and the CL_R_ was estimated by the glomerular filtration rate (GFR) method in PK-Sim^®^. Because there was no research that kidney transporters or tubules may be involved in the secretion, reabsorption and metabolism of PAZ, fraction of GFR was hence set at 1.0. Based on literature data (Minocha et al., 2012), it was thought transport of PAZ by efflux transporter BCRP1 at a PAZ concentration of 5°μM at an incubation time of between 60 and 120 min followed first-order kinetics. Hence, BCRP1 V_max_ was calculated to be 2.5 pmol/min by ratio of the cumulative transported amount to the corresponding hour, BCRP1 K_m_ was assumed to be 0.5 μM (1/10 of the substrate concentration).

**FIGURE 1:**
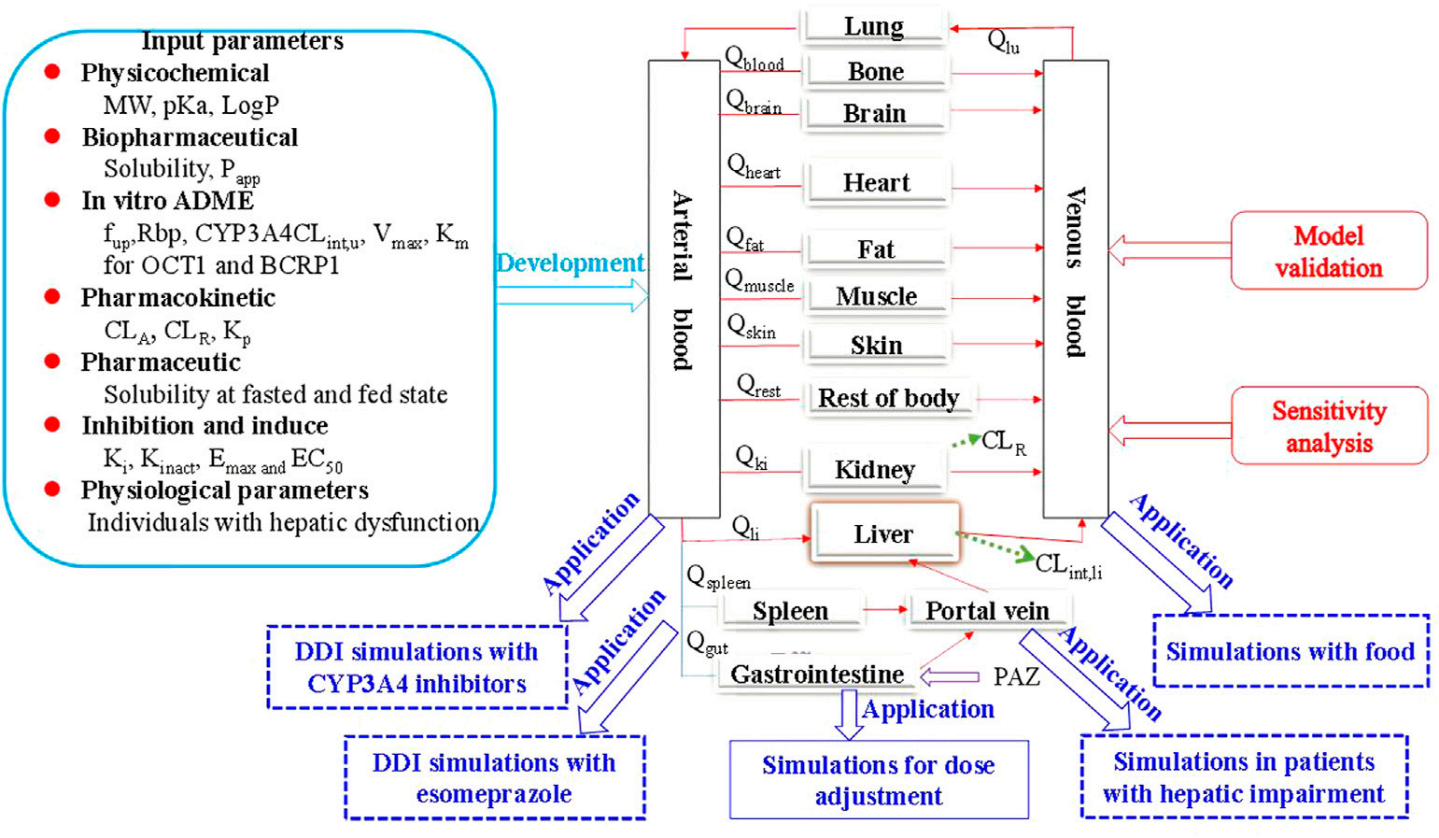
The generic workflow of the PBPK model for PAZ in human. The PBPK model is connected by blood flow rate (Q) and tissue compartments, which involves the gastrointestine, blood (arterial supply and venous return), eliminating tissues (liver and kidney) and non-eliminating tissues (13 compartments in total, such as the lung). The population PBPK model was built based on multiple modeling parameters and virtual population, and validated using the three PK profiles from the literature ((Deng et al., 2013) and (Heath et al., 2012)) and 11 different dosage regimen PK data sets from the literature ((Hurwitz et al., 2009)). Sensitivity analysis showed the two parameters are the most sensitive to the PBPK model. Subsequently, the PBPK model exhibited a wide application in five different aspects involving DDI with the two CYP3A4 inhibitors and with one acid-reducing agent, PK change with food, and PK change in patients with hepatic impairment. Finally, the PBPK model was used to determine the optimal dosing regimens under the above four clinical situations.

This PBPK model of PAZ was implicated in two metabolizing enzymes (CYP3A4 and UGT1A1) and six transporters (OCT1, OCT2, BCRP1, OATP1B1, MATE1, and MATE2K). Except for CYP3A4, reference concentrations of the others were not built into the PK-Sim^®^ expression database. Thus, it is needed to manually enter these data into PK-Sim^®^. Reference concentration of UGT1A1 was calculated by formula ((UGT1A1 abundance × 38 mg CYP protein/g liver)/liver volume) (Li et al., 2021), and calculated by formula ((transporter protein abundance × expressed organ weight)/liver volume) for OCT1, OCT2, and MATE1. Additionally, as MATE2-K protein expression was too low to be determined quantitatively, an alternative to this is to replace it with one-third of MRP4 (0.91 pmol/mg) expressed lowest in human kidney cortex (Prasad et al., 2016). Next, MATE2-K concentration in human kidney organ was calculated by formula (transporter expression × 26.2 mg/g × kidney weight) from the literature (Scotcher et al., 2017). The final inputting parameters used in PBPK model for PAZ are listed in Table 2 (Drozdzik et al., 2019; Li et al., 2019; Britz et al., 2020; Reddy et al., 2021; Krens et al., 2022; PMDA, 2022). The generic workflow of the PBPK model is represented in Figure 1.

**TABLE 2:** Summary of parameters used in PBPK model.

Property (Units)	Values used in the model	Literature values and source	Descriptions
MW(g·mol^−1^)	437.52	Chemspider	Molecular weight
pKa (base)	2.1, 6.4,10.2	(Food and Drug Administration, 2009)	Base dissociation constant
LogP	3.49 (mean value)	3.33 (Krens et al., 2022) and 3.65 (PMDA, 2022)	Lipophilicity
P_app_ (✕10^−6^ cm⋅s^−1^)	16.9	(Food and Drug Administration, 2009)	Caco-2 cell permeability
f_up_	0.00011	0.011% (Verheijen et al., 2017)	Fraction of free drug in plasma
Rbp	0.55	Calculated by PK-Sim	Blood-to-plasma concentration ratio
CYP3A4CL_int,u_ (μL/min/pmol)	1.10	Determined	Intrinsic clearance for CYP3A4
OCT1 V_max_ (pmol/min/mg protein)	530	530 pmol/min/mg protein (Ellawatty et al., 2018)	Maximum eflux velocity for OCT1
OCT1 K_m_ (μM)	3.47	3.47 μM (Ellawatty et al., 2018)	Michaelis-Menten constant for OCT1
BCRP1 V_max_ (pmol/min)	2.5	(Minocha et al., 2012)	Maximum eflux velocity for BCRP1
BCRP1 K_m_ (μM)	0.5		Michaelis-Menten constant for BCRP1
CL_R_(L/h)	GFR	—	renal clearance
GFR fraction	1.0	—	Fraction of filtered drug in the urine
CL_A_ (ml/min/kg)	0.04	Optimized	Additional systemic clearance
Partition coefficients	Rodgers and Rowland	Optimized	Calculation method from cell to plasma coefficients
Cellular permeabilities	PK-Sim Standard	Optimized	Permeability calculation method across cell
Reference concentration (μM/L liver tissue)	OCT1	0.077	64.2 fmol/mg liver (Drozdzik et al., 2019)
	BCRP1	0.045	105.8 fmol/mg small intestine (Drozdzik et al., 2019)
	UGT1A1	0.83	18.3 pmol/mg protein in liver (Reddy et al., 2021)
	OATP1B1	0.07	0.07 μM (Britz et al., 2020)
	OCT2	0.034	164.2 pmol/g kidney (Li et al., 2019)
	MATE1	0.022	105.6 pmol/g kidney (Li et al., 2019)
	MATE2-K	0.0049	0.91 pmol/mg protein (Prasad et al., 2016)
K_i_ CYP3A4 (μM)	4.0	4.0 μM (Yamada et al., 2020)	Inhibition constant at CYP3A4
K_inact_ CYP3A4 (min^−1^)	0.017	0.017 min^−1^ (Yamada et al., 2020)	The maximum rate of inactivation against CYP3A
E_max_ CYP3A4 (μM)	2.43	2.43 (Yamada et al., 2020)	Maximum inductive effect for CYP3A4
EC_50_ CYP3A4 (μM)	0.807	0.807 μM (Yamada et al., 2020)	Inducer concentration required to achieve 50% inductive effect
IC_50_ UGT1A1 (μM)	1.2	1.2 μM (Xu et al., 2010)	Inhibition constant at UGT1A1
IC_50_ OATP1B1(μM)	0.79	0.79 μM (Xu et al., 2010)	Inhibition constant at OATP1B1
K_i_ OCT2 (μM)	3.0	3.0 μM (Sauzay et al., 2016)	Inhibition constant at OCT2
K_i_ MATE1 (μM)	1.7	1.7 μM (Sauzay et al., 2016)	Inhibition constant at MATE1
K_i_ MATE2-K (μM)	3.3	3.3 μM (Sauzay et al., 2016)	Inhibition constant at MATE2-K

#### 2.5.2 PBPK model verification

The clinically observed PK profiles for PAZ (Heath et al., 2012; Deng et al., 2013) were first used to validate the predictive performance of this PBPK model. The prediction of the PBPK model was evaluated by the ratio between predicted and observed data. The common acceptable ratio is within 0.5–2.0. Next, PK variables of PAZ (AUC, C_max_, and C_trough_) from a clinical study (Hurwitz et al., 2009) under multiple dosage regimens were further used to verify the prediction of this PBPK model. The used modeling parameters and verified data are summarized in Table 3.

**TABLE 3:** List of the used modeling parameters and verified data.

Purpose	Modeling parameters	Parameters source	Verification data
Develop the PBPK model of PAZ	Physicochemical: MW, pKa, LogP	Chemspider, Literatures (Food and Drug Administration, 2009; Krens et al., 2022; PMDA, 2022)	(i) Verify under sing administration from Literatures (Heath et al., 2012; Deng et al., 2013) (ii) Verify under multiple dosage regimens from Literatures (Hurwitz et al., 2009)
	Biopharmaceutical: pH-solubility, P_app_	i) pH-solubility: experimentally determined	
		ii) P_app_: Literature (Food and Drug Administration, 2009)	
	*In vitro* ADME: f_up_, Rbp, CYP3A4CL_int_, V_max_, K_m_	i) CYP3A4CL_int_: experimentally determined	
		ii) Remaining parameters: Literatures (Minocha et al., 2012; Verheijen et al., 2017; Ellawatty et al., 2018)	
	Transporter concentration	Literatures (Prasad et al., 2016; Drozdzik et al., 2019; Li et al., 2019; Britz et al., 2020; Reddy et al., 2021)	
	Pharmacokinetic: CL_A_, CL_R_, K_p_	Optimized	
	Inhibition and induce: K_i_, K_inact_, E_max_, EC_50_	Literatures (Xu et al., 2010; Sauzay et al., 2016; Yamada et al., 2020)	
DDI simulation	PBPK modeling parameters of ketoconazole and lapatinib	See Supplementary Table S1, S2	Verify the effect of ketoconazole and esomeprazole on PK variables using the data form the literature (Tan et al., 2013)
	pH in stomach compartment after administration of esomeprazole	Literature (Rohss and Hedenstrom, 2002)	
Food effect simulation	Calories data of low-fat and high-fat food	Literature (Gajewska et al., 2020)	Verify at single dose of 800 and 600 mg PAZ OD, respectively, using the data form the literatures (Heath et al., 2010; Lubberman et al., 2019)
	Physiological parameters of gastrointestine in fasted and fed state	See Supplementary Table S3	
	pH-solubility data at fasted and fed state	Experimentally determined	
Hepatic impairment simulation	Physiological parameter in patients with impaired hepatic function	See Supplementary Table S5	Verify using the PK variables and profiles form the literature (Shibata et al., 2013)
	PPSF	Calculated using Eq. 4	

### 2.6 Sensitivity analysis

A sensitivity analysis was carried out to assess the effect of the used model parameters on the C_max_, C_trough_, and AUC after clinical dosing regimens (i.e., 800 mg OD) for PAZ, respectively. The parameters selected for the sensitivity analysis fulfilled the criteria: 1) optimized; 2) could have strong impact on the PK variables in this model. The effects of these examined parameters on C_max_, C_trough_, and AUC were assessed by altering the value of each parameter by±20% (Li et al., 2020). The calculation of sensitivity coefficient (SC) is given below (Li et al., 2020):

SC=ΔY/Y÷ΔP/P (3)

Where ∆Y represents the alteration of predicted C_max_, C_trough_, and AUC; Y is the initial value of predicted C_max_, C_trough_, and AUC; ∆P represents the alteration of assessed model parameters; P is initial value of assessed parameters. If certain SC value is more than 1.0 (i.e., it means that a 20% change of the assessed parameters results in a 10% alteration in C_max_, C_trough_, and AUC), it indicates this examined model parameter has significant influence on predicted C_max_, C_trough_, and AUC.

### 2.7 Virtual population demographic characteristics

Based on the demographic characteristics from each clinical study, information about the virtual population was set in PK-Sim^®^, including age range, body weight, height and proportion of female participants. If some data was absent, the mean value built in PK-Sim^®^ was used as a surrogate. Specifically, the PK variables and profiles of PAZ was simulated in 9 virtual population containing 3 females and 6 males, aged between 30 and 81 years, weight between 51.0 and 154.0 kg for the PBPK model construction and first validation based on the clinical PK study (Heath et al., 2012) in the fasting state. According to the population PK in a clinical study (Hurwitz et al., 2009), main PK varibales (AUC, C_max_ and C_trough_) under multiple dosing regimens were predicted in 63 virtual population containing 35 females and 28 males, aged between 40 and 73 years for further verification of the PBPK model in the fasting state.

### 2.8 DDI simulation

The developed PBPK model of PAZ was combined with the PBPK models of ketoconazole (a strong competitive CYP3A4 inhibitor) and lapatinib (a moderate competitive CYP3A4, P-gp and BRCP inhibitor), respectively, to simulate PK-DDIs between PAZ and CYP3A4 inhibitors. During DDI simulations, the dosage regimen was designed for PAZ as 400 mg OD (+ ketoconazole 400 mg OD), and for PAZ as 800 mg OD (+lapatinib 1,500 mg OD). Following administration of PAZ for 7 days, then co-administration of PAZ plus ketoconazole was simulated for another 5 days. DDI Simulations were conducted following PAZ with co-administration of CYP3A4 inhibitors in 21 virtual population containing 11 females and 10 males, aged between 37 and 80 years at fasting state (Tan et al., 2013), respectively. The final inputting parameters used in the PBPK model for the two modulators are listed in Supplementary Tables S1, S2.

Based on a clinical DDI study about the concomitant use of esomeprazole and PAZ (Tan et al., 2013), simulations were designed that on day 7, when patients received PAZ 800 mg OD, they began to take esomeprazole for consecutive 5 days, followed by DDI estimation between two drugs. Moreover, from a literature study (Röhss and Hedenström, 2002), after esomeprazole was administered orally at a 40 mg dose OD for a total of 5 days, human intragastric mean 24-h pH was 4.78. Thus, pH in stomach compartment in PK-Sim^®^ was manually modified as 4.78 and loaded with experimental solubility data at pH = 4.78 in stomach comparment (Table 1) into this PBPK model to simulate the effect of elevated intragastric pH on the PK alteration of PAZ. During simulation, the dosage regimen was designed for PAZ as 800 mg OD for consecutive 12 days. Simulation for influence of esomeporazole on PK variables of PAZ was conducted in 13 virtual population containing 11 females and 2 males, aged between 36 and 75 years in the fasting state.

### 2.9 Food effect simulation

According to the published papers (Gajewska et al., 2020), Supplementary Table S3 summarizes physiological parameters used in the human gastrointestinal tract in fasted and fed states. The influence of food on the PK variables of PAZ was conducted by adjustment of gastrointestinal physiological parameters (Supplementary Table S3) and by loading pH-solubility data in fed test media (Table 1) into the current PBPK model. Based on a clinical study (Heath et al., 2010), simulations were carried out after PAZ was administered at a single 800 mg OD with food containing low-fat and high-fat meals, and without food for consecutive 14 days, respectively. Referring to relevant literature (Gajewska et al., 2020), low-fat and high-fat food were set to contain 330 and 1,000 cal in this simulation, respectively. The simulations were carried out in 16 virtual population containing 7 females and 9 males, aged between 36 and 76 years for population eating high-fat food and 13 virtual population containing 8 females and 5 males, aged between 36 and 77 years for population eating low-fat food, respectively (Heath et al., 2010). Based on another clinical study (Lubberman et al., 2019), simulations were carried out after PAZ was administered at single doses of 800 and 600 mg OD in 60 virtual population containing 16 females and 44 males, aged between 28 and 85 years for population receiving standard breakfast.

### 2.10 Hepatic impairment simulation

In this simulation, the used physiological parameters in patients with hepatic impairment were taken from the published papers (Heimbach et al., 2021; Willmann et al., 2021). The plasma protein scale factor (PPSF) in PK-Sim^®^ was modified to describe changes in plasma albumin protein concentration and unbound PAZ fraction (Gerner and Scherf-Clavel, 2021). Based on the following equation derived from the paper (Johnson et al., 2010), PPSF was estimated.

$$PPSF=\frac{1}{f_{up}+\left(1-f_{up}\right)\times {Albumin}_{f}}$$ (4)

Where Albumin_f_ is the fractional value of plasma albumin in patients with hepatic impairment with respect to healthy individuals. The gastrointestinal solubility in patients with liver dysfunction was assumed to be reduced to 80, 50 and 20% of corresponding values in individuals with normal liver function, respectively. In addition, simulations for PK variables and profiles of PAZ in patients with liver dysfunction were conducted in 14 virtual population containing 6 females and 8 males, aged between 47 and 78 years, 13 virtual population containing 9 females and 4 males, aged between 36 and 76 years, and 19 virtual population containing 7 females and 12 males, aged between 39 and 78 years for mild, moderate, and severe hepatic impairment, respectively. The final physiological parameters used in this model in patients with hepatic impairment relative to a mean individual with normal hepatic function are summarized in Supplementary Table S4.

## 3 Results and discussion

### 3.1 *In vitro* assays

The experimentally determined solubility in dependence of pH is shown in Table 1. When the pH was greater than 3.0, the solubility of PAZ decreased significantly with an increase in the pH. The solubility cannot even be detected when pH ≥ 7.0. On the other hand, intestinal solubility of PAZ after a meal relative to a fasted state increased approximately by 10-fold from 0.63 μg/ml in FaSSIF at pH 6.5 to 6.5 μg/ml in FeSSIF at pH 5.8. Additionally, as described in Supplementary Table S5, the solubility sharply increased by about 4-fold as the NaTC rose from the fasted to the fed state concentration (15 mM compared with 30 mM).Therefore, PAZ absorption can be influenced by the combined effect of human gastrointestinal pH and bile salt release.

The depletion of PAZ over time by human recombinant CYP3A4 was determined for assessing the metabolism contribution of CYP3A4 enzyme. The experimental result is presented in Supplementary Figure S1. As shown in Supplementary Figure S1, only about 30% of PAZ was depleted during 90 min, indicating that it was to a modest extent metabolized by CYP3A4. The estimated mean k and CL_int_ for PAZ were 0.0053 min^−1^ and 1.10 μl/min/pmol, respectively (see Supplementary Table S6).

### 3.2 Development and validation of PBPK model in humans

The PBPK model for PAZ was developed using a large number of parameters in Table 1 and Table 2. Typically, gastric emptying time in normal individuals is in the range of 0.25–0.5 h. To reduce the difference between predicted and observed T_max_, the gastric emptying time was set at 0.4 h in the fasted simulation (Supplementary Table S3). The gastrointestinal pH at a fasted state was set using data in Supplementary Table S3 from the literature (Gajewska et al., 2020). In general, gastric pH in fasted state is within 1.3–2.0 (Giarratano et al., 2018). To better predict multiple PK variables from the literatures (Hurwitz et al., 2009; Heath et al., 2012), gastric pH was set using a mean value of 1.6 in this simulation at fasted state. The solubility data in fasted state (see Table 1) were introduced into the PBPK model to simulate human PK profiles of PAZ without food.

**FIGURE 2:**
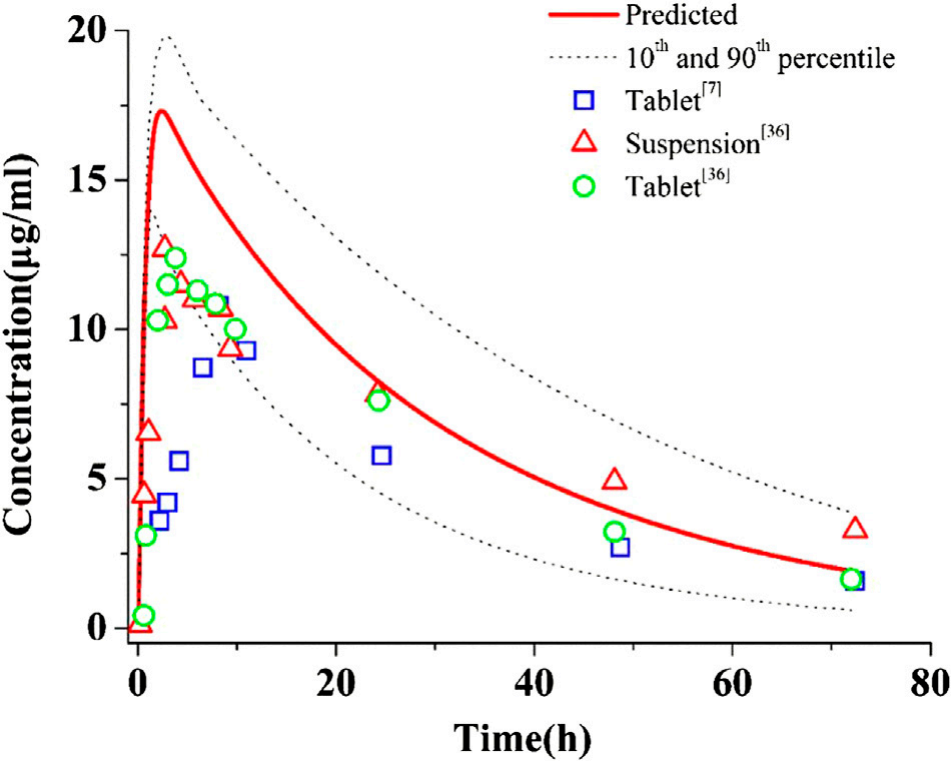
Simulations of pharmacokinetics of PAZ in humans after an oral administration of 400 mg single dose. The blue squares (□), red up-triangles (△) and green circles (○) refer to clinically measured pharmacokinetic data of PAZ tablet and suspension from references 7 and 36.

**FIGURE 3:**
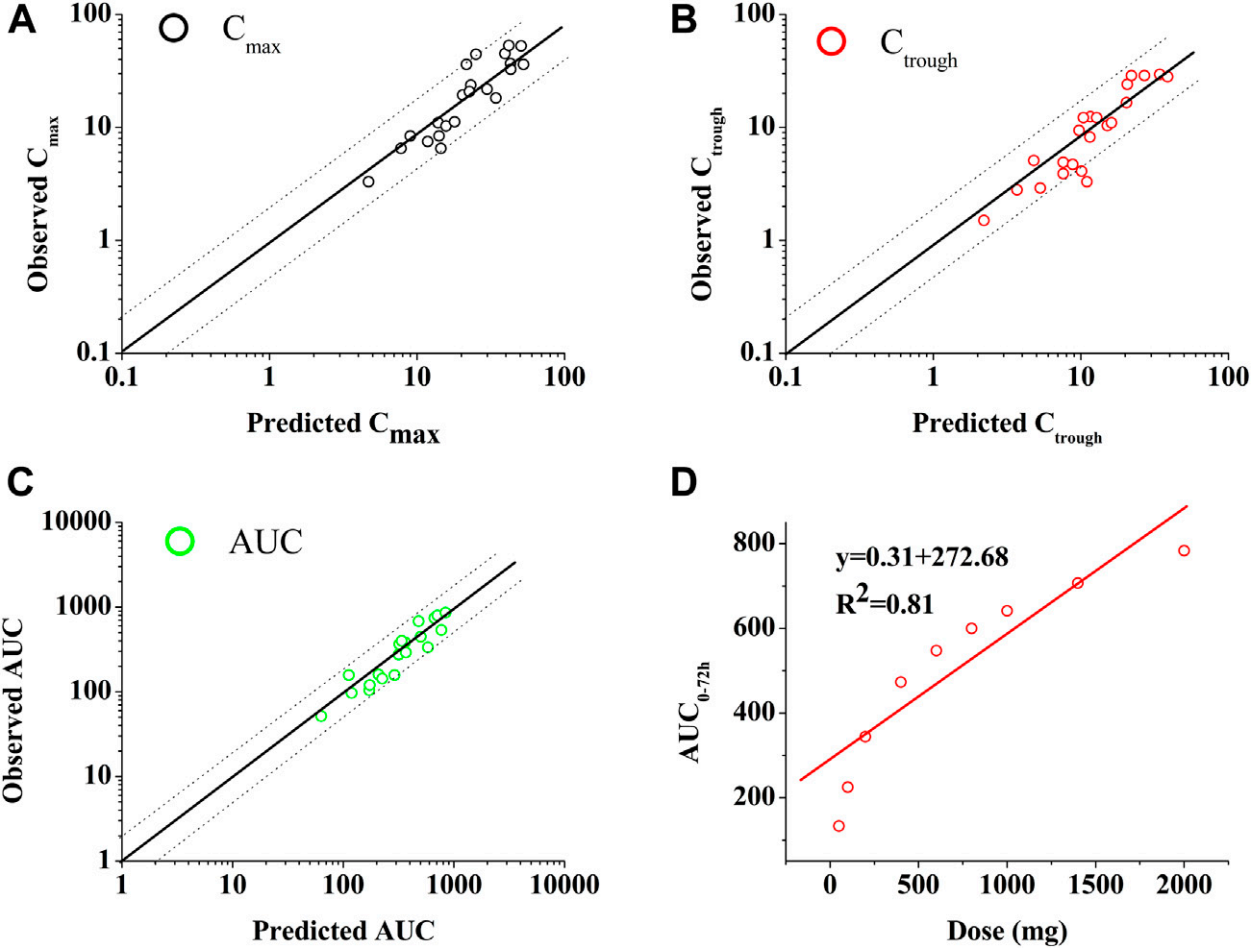
Goodness-of-fit plot of the PBPK mode of PAZ for predicted and observed C_max_ **(A)**, C_trough_ **(B)** and AUC **(C)** and relationship between dose and AUC **(D).** The identity line and acceptable limits (0.5–2.0 fold) are shown as solid and dashed lines, respectively. **(A,B,C)** The black circles (○), red circles (○) and green circles (○) represent the ratios of C_max_, C_trough_ and _AUC_, respectively, between prediction and observation. **(D)** The data (○) represents the predicted values using the PBPK model. The red solid line via the data points reflects the best fit by the line equation.

Figure 2 shows the predictions and observations of plasma concentration-time profiles after a single-dose administration of 400 mg of PAZ. The comparison of arithmetic mean PK variables between predicted and observed data is shown in Table 4. As shown in Figure 2, the developed population PBPK model can basically capture the three clinically determined PK profiles. Next, the PK variables of PAZ were simulated following administration of multiple ascending doses (MAD) to further verify the predictive power of this model. The ratios of predictions and observations are shown in Supplementary Table S7. The 96% of predicted/observed C_max_ ratios, 91% predicted/observed C_trough_ ratios and all predicted/observed AUC ratios were within 0.5–2.0 (Supplementary Table S7). For clinical therapeutic dose of 800 mg, above three predicted/observed ratios were in the range of 0.73–1.15. Figure 3 plotted from all the data in Supplementary Table S7 further visually confirmed a good fit of the PBPK model of PAZ for nearly all clinical data under the MAD. Furthermore, the PBPK model confirmed that AUC of PAZ increase in a less than dose-proportional fashion as the oral dose rose from 50 to 2000 mg, especially more than 200 mg (Figure 3D). The relationship of AUC and dose was fitted as AUC = 0.31 × dose. The slope is close to the clinically determined value, 031 compared with 0.46 (Food and Drug Administration, 2009).

**TABLE 4:** Comparisons of PK variables (arithmetic mean, range) between predicted and observed data.

Clinical study	PK variables	Prediction	Observation	Prediction/observation ratio
400 mg tablet^a^ (Deng et al., 2013)	C_max_ (μg mL^−1^)^c^	17.3 (14.0–19.9)	10.8 (7.3–12.5)	1.60
	C_24_ (μg mL^−1^)^d^	8.3 (4.6–12.0)	5.8 (NC)	1.43
	AUC_0-72_ (μg h mL^−1^)^e^	503.1 (290.1–718.2)	335.2 (259–582)	1.50
	T_max_(h)	2.3 (1.4–2.8)	8.0 (4.0–10.0)	0.29
400 mg oral suspension^b^ (Heath et al., 2012)	C_max_ (μg mL^−1^)	17.3 (14.0–19.9)	12.7 (NC)	1.36
	C_24_ (μg mL^−1^)	8.3 (4.6–12.0)	7.8 (NC)	1.06
	AUC_0-72_ (μg h mL^−1^)	503.1 (290.1–718.2)	471.1 (NC)	1.07
	T_max_(h)	2.3 (1.4–2.8)	2.7 (NC)	0.85
400 mg whole tablet^b^ (Heath et al., 2012)	C_max_ (μg mL^−1^)	17.3 (14.0–19.9)	12.4 (NC)	1.40
	C_24_ (μg mL^−1^)	8.3 (4.6–12.0)	7.6 (NC)	1.09
	AUC_0-72_ (μg h mL^−1^)	503.1 (290.1–718.2)	410.3 (NC)	1.23
	T_max_(h)	2.3 (1.4–2.8)	3.8	0.61

a : Data were taken from reference 7.

b : Data were taken from reference 36.

c : C_max_ is peak concentration of PAZ.

d : C_24_ is PAZ, concentration at 24 h time point.

e : AUC_0-72_ is the area under plasma concentration vs time (0–72 h) curve.

NC: not calculated.

### 3.3 Sensitivity analysis

**FIGURE 4:**
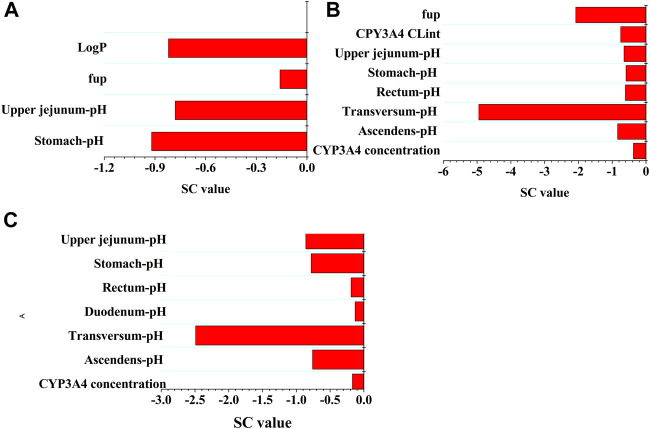
Sensitivity analysis of the PBPK model. The parameter sensitivity of the PBPK model to single parameter is measured with the change of the predicted C_max_ **(A)**, the predicted C_trough_ **(B)**, and AUC **(C)**.

A sensitivity analysis was performed at a therapeutic 800 mg oral dose to evaluate the influence of the selected parameters on the PBPK model. The PBPK model was the most sensitive to gastric pH (SC: 0.92) and Log P (SC: −0.82) for C_max_, pH in the colon (SC: −4.95) and f_up_ (SC: −2.08) for C_trough_, and pH in the colon (SC: −2.49) and pH in the jejunum (SC: −0.86) for AUC, respectively. Also of note was that only two SC values for C_trough_ and one SC value for AUC were greater than 1.0. In summary, sensitivity analysis indicated the majority of modeling parameters had minor impact on the three PK variables of PAZ. The sensitivity analysis is given in Figure 4.

### 3.4 Model application

#### 3.4.1 DDI simulations

**FIGURE 5:**
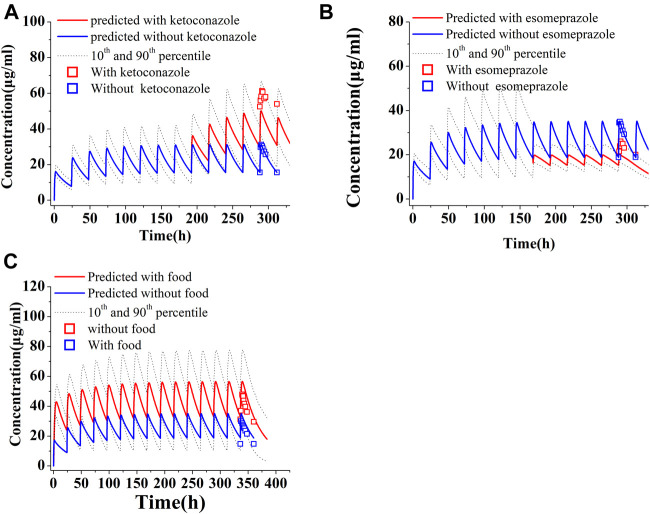
Simulations of pharmacokinetics of PAZ under different situations. **(A)** PAZ 400 mg OD was first administered for 7 days, then co-administration of PAZ plus ketoconazole (400 mg OD) was given for another 5 days. **(B)** PAZ 800 mg OD was administered for 7days, then co-administration of PAZ plus esomeprazole (40 mg OD) was given for another 5 days. **(C)** Co-administration of PAZ 800 mg OD with food were simulated for consecutive 14 days. The blue squares (□) refer to clinically measured values without ketoconazole **(A)**, esomeprazole **(B)** and food **(C)**. The red squares (□) refer to clinically measured values with ketoconazole **(A)**, esomeprazole **(B)** and food **(C)**.

The predicted and observed PK variables and profiles of two CYP3A4 inhibitors (ketoconazole and lapatinib) have been given in Supplementary Table S8 and Supplementary Figure S2. The predicted PK profile of PAZ with ketoconazole is shown in Figure 5A. Although DDI simulations with ketoconazole underestimated the observed data, the 90% prediction interval almost covered the variability of the observed data. The PK-DDI ratios predicted by the PBPK model are summarized in Table 5. The C_max_, C_trough_, and AUC_288-312_ ratios of 400 mg PAZ co-administrated with ketoconazole were approximately 1.60, 1.96, and 1.59-fold higher, respectively, than PAZ alone. The impact of 1,500 mg lapatinib on the PK variables of PAZ was similar to that of ketoconazole (see Table 5). When co-administration with the two CYP3A4 inhibitors, the predicted C_max_, C_trough_ and AUC_288-312_ ratios of PAZ were quite close to the observed data (Food and Drug Administration, 2009; Tan et al., 2013).

**TABLE 5:** PK variable changes (arithmetic mean, range) of PAZ with or without co-administration of other drugs.

Variables	PAZ only^d^ (400 mg)	PAZ + ketoconazole^e^ (400 mg, OD)	Predicted ratio	Observed ratio
C_max_ (μg·mL^−1^)^a^	31.3 (23.5–41.9)	50.1 (36.9–67.0)	1.60	1.45
C_trough_ (μg·mL^−1^)^b^	15.7 (9.6–23.0)	30.8 (18.4–46.5)	1.96	1.81
AUC_288-312_ (μg·h·mL^−1^)^c^	555.5 (387.6–874.3)	882.0 (616.3–1,208.4)	1.59	1.66

Variables	ACA only^d^ (800 mg)	PAZ + Lapatinib^e^ (1,500 mg, OD)	Predicted ratio	Observed ratio
C_max_ (μg·mL^−1^)	31.3 (23.5–41.9)	46.5 (33.6–61.6)	1.49	1.51
C_trough_ (μg·mL^−1^)	15.7 (9.6–23.0)	26.7 (15.9–36.4)	1.70	—
AUC_288-312_ (μg·h·mL^−1^)	555.5 (387.6–874.3)	873.7 (583.3–1,183.3)	1.57	1.59

Variables	ACA only^d^ (800 mg)	PAZ + Esomeprazole^e^ (40 mg, OD)		Ratio
C_max_ (μg·mL^−1^)	35.2 (21.2–48.5)	19.8 (16.6–24.6)	0.56	0.58
C_trough_ (μg·mL^−1^)	19.1 (8.9–31.1)	15.3 (12.4–20.0)	0.80	0.64
AUC_288-312_ (μg·h·mL^−1^)	723.2 (516.1–1,032.5)	491.1 (404.3–616.8)	0.68	0.60

^a,b^C_max_ and C_trough_ is peak concentration (a) of PAZ, and trough concentration (b) at steady state, respectively.

c AUC_288-312_ is the area under plasma concentration vs time (288–312 h) curve.

d Single oral administration.

e Multiple oral administration and determined at 13th day.

Another simulation is toward to the PK change of PAZ when concomitant use with esomeprazole. As shown in Figure 5B, the predicted PK profile cannot sufficiently reproduce the observed profile with an underestimation, however, the 90% prediction interval almost covered the variability of the observed profile. Instead, there was less difference between predicted and observed ratios, with predicted ratios of 1.49, 1.70, and 1.57 for C_max_, C_trough_ and AUC_288-312_, respectively. The predicted ratios with esomeprazole are also in good agreement with the clinically observed data (Tan et al., 2013).

#### 3.4.2 Impacts of food on PK variables of PAZ

The gastrointestinal pH, transmit time, and stomach volume after a high-fat and low-fat meal were set using the data in Supplementary Table S3, respectively. The fed-state solubility data (see Table 1) were introduced into the PBPK model to simulate human PK profiles of PAZ with food. It should be noted that the mean value (166.7 μg/ml) of the three solubility in the FEGGAS medium was used to simulate the effect of food on the PK variables of PAZ.

The PK variables of PAZ were first simulated at a single 800 mg dose when given with high and low-fat food, respectively. As seen in Table 6, the PK variables of PAZ with food were increased by 2-3 fold. Slight overestimations were found compared with observed ratios (Heath et al., 2010) (C_max_ 2.30 vs. 2.10 and AUC_0-72_: 2.65 vs. 1.92). Additionally, it was also found that the effect of low-fat food on the PK variables of PAZ is nearly the same as those when given high-fat food.

**TABLE 6:** PK variable (geometric mean, range) changes of PAZ with or without food.

Variables	PAZ only^a^	PAZ (800 mg)+Low-fat^b^	Predicted ratio	Observed ratio
C_max_ (μg·mL^−1^)	21.0 (17.2–25.5)	48.4 (37.7–59.4)	2.30	2.10
C_trough_ (μg·mL^−1^)	10.3 (6.9–12.1)	30.1 (22.5–39.6)	2.92	—
AUC_0-72_ (μg·h·mL^−1^)	624.3 (416.1–832.5)	1,654.4 (1,127.9–2313.6)	2.65	1.92

Variables	PAZ only^a^	PAZ (800 mg)+High-fat^b^	Predicted ratio	Observed ratio
C_max_ (μg·mL^−1^)	21.8 (17.9–24.6)	51.0 (40.0–71.2)	2.34	2.08
C_trough_ (μg·mL^−1^)	10.9 (7.3–13.6)	32.7 (22.2–42.3)	3.00	—
AUC_0-72_ (μg·h·mL^−1^)	685.9 (489.1–906.3)	1766.7 (1,179.2–2534.0)	2.58	2.34

Variables	ACA only^b^	PAZ (600 mg)+Low-fat^b^	Predicted ratio	Observed Ratio
C_max_ (μg·mL^−1^)	38.5 (30.6–49.1)	56.1 (34.5–77.6)	1.46	1.09
C_trough_ (μg·mL^−1^)	17.5 (9.3–33.9)	26.8 (10.7–52.8)	1.53	1.12
AUC_336-360_ (μg·h·mL^−1^)	713.5 (503.6–1,039.5)	994.8 (533.1–1,593.3)	1.39	1.10

a : Single oral administration.

b : Repeat daily dosing for consecutive 14 days.

Another simulation was carried out after repeat daily dosing of PAZ at a 600 mg dose for consecutive 14 days with low-fat food. Figure 5C suggests that the PBPK model is able to sufficiently capture the clinically determined PK profiles. In this simulation, the prediction/observation ratios were 1.40 for C_max_, 1.53 for C_trough_ and 1.39 for AUC_336-360_, respectively. These predicted ratios were slightly higher than the observed data (Lubberman et al., 2019) (see Table 6).

#### 3.4.3 Simulations in patients with hepatic impairment

The corresponding physiological parameters were manually adjusted in PK-Sim^®^ using data from Supplementary Table S4. Meanwhile, the PPSF and gastrointestinal solubility (Supplementary Table S4) were entered into PK-Sim^®^ to replace the corresponding data in individuals with normal hepatic function.

**FIGURE 6:**
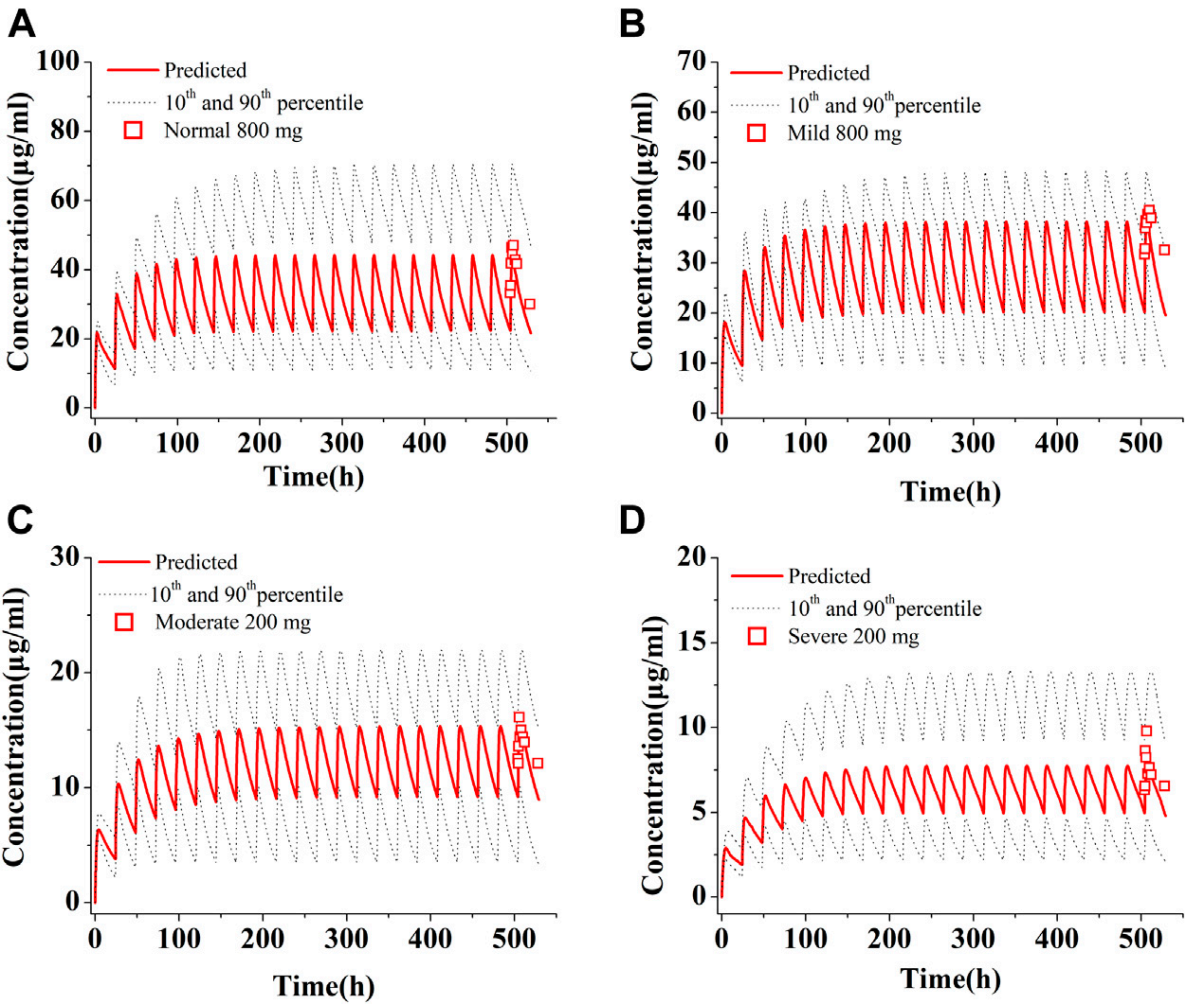
Simulations of pharmacokinetics of PAZ in patients with normal hepatic function **(A)**, with mild **(B)**, moderate **(C)** and severe **(D)** hepatic impairment. The red squares (□) refer to clinically measured values in patients with normal hepatic function at 800 mg OD **(A)**, with mild hepatic impairment at 800 mg OD **(B)**, and with moderate **(C)** and severe **(D)** hepatic impairment at 200 mg OD. All simulations were run at repeated daily doses for a consecutive 21 days.

The population simulations were conducted for PAZ in patients with hepatic impairment under repeated daily doses of 800 mg (normal and mild) and 200 mg (moderate and severe) for consecutive 21 days Figure 6 shows predicated and observed PK profiles in patients with hepatic impairment. Predicted and observed ratios of PK variables are given in Table 7. The simulations showed that the predicted results were consistent with the observed PK variables (Shibata et al., 2013). As described in Table 7, the median steady-state PAZ C_max_, C_trough_ and AUC_504-528_ were slightly decreased for mild hepatically impaired patients and meaningfully reduced for moderate and severe hepatically impaired patients. After oral administration of 800 mg OD, The median PK variables in patients with mild hepatic impairment were above 80% of those in patients with normal hepatic function. The median PK variables after administration of 200 mg PAZ OD in patients with moderate hepatic impairment were 31% (C_max_), 39% (C_trough_) and 34% (AUC_504-528_) of the corresponding median data following 800 mg PAZ in patients with normal hepatic function, respectively. The three median PK variables in patients with severe hepatic impairment were only about 20% of the corresponding median data in patients with normal hepatic function.

**TABLE 7:** PK variable (Median) ratios of PAZ in hepatic impairment patients.

Variables	Ratio^a^					
	Predicted			Observed		
	Mild (800 mg)	Moderate (200 mg)	Severe (200 mg)	Mild	Moderate	Severe
C_max_ (μg·mL^−1^)	0.81	0.31	0.15	0.64	0.43	0.18
C_trough_ (μg·mL^−1^)	0.82	0.39	0.16	0.81	0.54	0.19
AUC_504-528_ (μg·h·mL^−1^)	0.83	034	0.17	0.87	0.30	0.15

a : Calculated by dividing normal data (800 mg) with mild, moderate and severe, respectively.

#### 3.4.4 Dosing adjustment recommendation based on the PBPK model

The established strategy of dosing adjustment was mainly according to the ratio change of AUC_0-t_. However the dose under-proportional property of PAZ (AUC = 0.31 × dose) suggests that the dosing modification regimen cannot be straightforward. On the other hand, based on clinical PK-efficacy studies, to attain sufficient clinical response, C_trough_ of PAZ is needed to achieve above 15 μg/ml (Food and Drug Administration, 2009), even >20 μg/ml (Verheijen et al., 2017). Therefore, a minimal of 15 μg/ml concentration for the C_trough_ threshold of clinical efficacy is defined. In addition, based on the clinical exposure-toxicity study (Food and Drug Administration, 2009), it is more optimum dose when C_max_ and C_trough_ is less than 56 μg/ml and 32 μg/ml, respectively. Hence, in this work, it was considered a better strategy to adjust dosage regimen of PAZ under various clinical situations using the combination of the PBPK simulation with the threshold of clinical efficacy and safety. Table 8 summarized our simulations for dosing modification recommendations using the combination strategy.

**TABLE 8:** PAZ dosing adjustment recommendation based on the PBPK model.

Scenario	Arithmetic mean at steady state (90% CI)			Based-model recommendation
	C_max_ (μg·mL^−1^)	C_trough_ (μg·mL^−1^)	AUC_504-528_ (μg·h·mL^−1^)	
DDI (PAZ + Ketoconazole 400 mg OD)				
100 mg OD	17.4 (12.5–27.4)	9.8 (5.7–14.0)	177.2 (119.0–241.5)	Supports dose reduction to 200 mg OD
100 mg BID^a^	29.8 (20.0–41.0)	24.0 (14.4–32.5)	166.7 (111.5–229.1)	
200 mg OD	31.5 (22.4–42.7)	19.4 (11.1–25.8)	454.1 (310.3–621.6)	
400 mg OD	50.1 (36.9–67.0)	30.8 (18.4–41.7)	687.0 (480.1–941.3)	
800 mg OD	66.5 (50.5–87.9)	41.9 (21.9–51.2)	892.3 (632.9–1,221.4)	
DDI (PAZ + Lapatinib 1,500 mg OD)				
100 mg OD	16.7 (11.6–25.8)	10.3 (5.3–16.9)	323.1 (211.8–368.2)	Support dose reduction to 200 mg OD
200 mg OD	30.9 (19.6–38.1)	18.5 (12.6–27.1)	533.7 (353.7–768.9)	
400 mg OD	49.4 (33.5–56.8)	29.1 (13.0–38.9)	823.7 (513.2–1,102.6)	
800 mg OD	65.5 (58.6–78.1)	39.3 (32.6–47.5)	1,079.9 (873.2–1,362.1)	
DDI (PAZ + Esomeprazole 40 mg OD)				
800 mg OD	19.8 (16.6–24.6)	15.3 (12.4–19.9)	491.1 (404.3–616.8)	No need to adjust dose
1,200 mg OD	22.9 (19.1–27.6)	17.2 (13.8–22.2)	504.4 (427.6–645.9)	
1,600 mg OD	25.4 (21.2–30.0)	18.7 (14.9–23.9)	554.1 (468.1–703.0)	
2000 mg OD	27.6 (23.0–33.0)	20.6 (15.9–25.3)	597.6 (503.7–754.2)	
With Food				
100 mg OD	15.2 (12.1–20.0)	7.6 (4.7–12.6)	425.3 (312.0–597.6)	Support dose adjustment to 100 mg BID
100 mg BID	26.1 (20.4–35.9)	19.7 (13.6–29.3)	372.4 (277.9–537.0)	
200 mg OD	29.3 (23.0–38.8)	15.5 (9.9–25.4)	837.7 (615.1–1,180.3)	
400 mg OD	52.9 (3.5–71.2)	29.4 (18.6–50.2)	1,538.3 (1,046.0–2238.8)	
Hepatic Impairment				
Mild	400 mg	28.8 (21.4–37.9)	14.6 (7.7–24.3)	527.3 (336.4–734.2)
	600 mg	33.8 (27.3–42.8)	16.4 (9.2–27.9)	600.0 (416.3–834.0)
	800 mg	44.2 (29.8–48.3)	22.3 (9.7–35.0)	782.3 (508.2–1,059.8)
Moderate	200 mg OD	13.8 (7.6–34.7)	8.6 (3.5–15.3)	263.9 (142.8–399.8)
	200 mg BID	30.8 (14.9–40.3)	24.6 (9.1–34.9)	439.8 (194.4–627.9)
	400 mg OD	20.7 (12.3–29.7)	13.9 (4.6–20.1)	405.5 (198.0–597.5)
	800 mg OD	21.9 (14.7–36.3)	14.4 (5.6–25.6)	430.0 (242.2–734.2)
Severe	PAZ 200 mg	7.7 (4.8–20.9)	4.9 (2.7–9.3)	151.6 (80.8–266.6)
	PAZ 400 mg	9.8 (5.9–17.0)	6.3 (2.8–12.2)	192.9 (102.9–344.7)
	PAZ 800 mg	11.9 (7.1–20.6)	7.9 (4.3–15.7)	236.1 (125.2–424.9)

a : twice daily.

##### 3.4.4.1 Simulations of PAZ dosing adjustment when co-administered with CYP3A4 inhibitors

Despite the fact that PAZ undergoes a moderate metabolism (about 30% depletion) by CYP3A4, given that there is a small difference between effective and toxic plasma concentration, the simulations for dosing adjustment to overcome the DDI are still necessary. Due to a lack of metabolism data of PAZ by CYP3A4, the substrate depletion method was used to experimentally determine the clearance mediated by CYP3A4 (see Supplementary Table S6). The co-administration with ketoconazole and lapatinib resulted in a less than 2-fold increase in PK variables compared to PAZ alone, which did not appear to need administration dose adjustment. However, simulated C_max_ and C_trough_ for the 800 mg OD dose regimen are both above the threshold of side effect (C_max_ <56 μg/ml; C_trough_<32 μg/ml), while C_max_ and C_trough_ for the 100 mg BID and 200 mg OD dose regimens are within clinical effective ranges (C_trough_ >15 μg/ml) (see Table 8).

The PBPK simulations of dosage adjustment of PAZ suggested that 200 mg OD or 100 mg BID can represent a suitable dosing regimen when co-administered with the two CYP3A4 inhibitors. Nevertheless, in consideration of only the approved tablet in strength of 200 mg and patient compliance (reduce the frequency of administration), 200 mg OD represents an optimal dosing regimen for PAZ clinical use when co-administered with CYP3A4 inhibitors.

##### 3.4.4.2 Simulations of PAZ dosing adjustment under the situations of gastrointestinal pH changes

Because PAZ has a very poor and pH-dependent aqueous solubility, changes in gastrointestinal pH can have a significant influence on the absorption of PAZ when co-administration with acid-reducing agents (such as esomeprazole) or with food. The effect of food on the PK variables of drugs that have the pH-dependent solubility property is a common phenomenon. According to literature (Riedmaier et al., 2020), this incidence is as high as 40% for orally administered drugs. Previous research (Budha et al., 2012) revealed that the gastrointestinal pH changes will have a significant impact on the PK variables of a drug when a drug meets the two criteria: 1) marked solubility decreasing in the pH range of 1–4; and 2) maximum dose strength being insoluble in 250 ml of aqueous media at above gastric pH. PAZ meets the above two criteria. Hence, this PBPK model can also be used to assess the gastrointestinal pH on the PK variables of PAZ. When using the single solubility or simple pH-solubility data from the literature to develop the model (Sugihara and Taylor, 2018; Fink, 2020), it was found that the model was unable to predict PK variable changes of PAZ co-administered with esomeprazole or with food. Instead of this, the solubilities in the multiple different pH conditions and the biorelevant media were experimentally determined (see Table 1). The solubility data reflect PAZ’s true gastrointestinal solubility of when given in tablet form. Therefore, this developed PBPK model using the experimentally determined solubility is able to reproduce clinically determined PK profiles of PAZ when co-administered with esomeprazole or with food (Figure 5; Tables 5, 6).

When co-administered with esomeprazole the simulated PK variables are comparable across the four dosage groups (Table 8). This can be explained by low near saturated absorption induced by a reduced gastric pH. Based on the PBPK simulation, 800 mg OD can be a suitable dosing regimen. On the other hand, when co-administered with food, the simulated C_max_ and C_trough_ for the 100 mg BID dose regimen are both within the threshold of clinically effective and side effect ranges. Hence, 100 mg BID can represent a suitable dosing regimen. Nevertheless, in consideration of only the approved tablet in strength of 200 mg, it could be a better dosing strategy to retain the unchanged 800 mg OD dosing regimen, when avoiding taking PAZ with food. This is in accordance with the PAZ label.

##### 3.4.4.3 Simulations of PAZ dosing adjustment in patients with hepatic impairment

Because hepatic impairment can significantly alter a patient’s physiology, parameters adjustment in absorption, distribution, metabolism, and elimination process for patients with hepatic impairment need to be considered in the development of PBPK model. It has been well known that the change of physiological processes in distribution, metabolism, and elimination occurs. Currently, these parameter changes have been applied to multiple PBPK models (see Supplementary Table S4). However, most published papers for PBPK models of hepatic impairment have not taken into account absorption changes between impaired and normal hepatic function (Morcos et al., 2018; Gerner and Scherf-Clavel, 2021; Fan et al., 2022).

A previous study confirmed that bile salt concentration is also changed when hepatic impairment occurs (Turnberg and Grahame, 1970). Recent studies have revealed that most current PBPK models are found to typically have an overestimation of PK in patients with moderate and severe hepatic impairment (Morcos et al., 2018; Heimbach et al., 2021). This could be explained by the fact that existing PBPK models cannot account for changes in absorption (especially poor soluble drug) induced by hepatic disease (Heimbach et al., 2021). In absorption process, for poorly soluble PAZ, the impact of reduced bile salt concentration on PAZ gastrointestinal solubility might be huge. However, so far, experimentally determined bile salt concentrations in patients with hepatic impairment have not been reported yet. In this simulation, it was assumed that PAZ solubility was reduced by 20, 50, and 80% in patients with mild, moderate, and severe hepatic impairment due to decreased bile salt excretion. In addition, another parameter change needs to be pointed out. Despite a number of studies have describe alterations in plasma protein concentration in liver disease, different estimated PPSFs (see Supplementary Table S4) were used to describe these changes for different hepatically impaired stages in this simulation.

The simulated C_max_ and C_trough_ values in patients with hepatic dysfunction are both within the efficacy and safety thresholds for the 600 and 800 mg OD dose regimens in patients with mild hepatic impairment and for the 200 mg BID dose regimen in patients with moderate hepatic impairment. Hence, dosage adjustment cannot be required for mild hepatic impairment, and the 20 mg BID represents a suitable dosing regimen for patients with moderate hepatic impairment. Additionally, the C_trough_ value in patients with severe hepatic impairment for all dose regimens is below efficacy threshold. As a result, PAZ is not recommended for use in the clinic for patients with severe hepatic impairment. Except for the recommended dosage in patients with moderate hepatic impairment, the other two recommended clinical uses by this PBPK model are consistent with clinical study (Food and Drug Administration, 2009). The clinical dosage recommendation in patients with moderate hepatic impairment (200 mg OD) possibly results from the combined results of a PK study in patients (Shibata et al., 2013) (almost equal median C_max_ and C_trough_ were observed in 200 and 400 mg groups) and clinical safety.

In general, if AUC of a drug PK compared to use alone increases or decreases by about 2-fold, clinical dosage adjustment should be considered. However, due to the dose under-proportional property and the small difference between effective and toxic plasma concentrations of PAZ, the modification of dosing regimen cannot be straightforward. In this work, it was suggested that it can be a superior option for dosing adjustment in various clinical situations based on this PBPK model. A prominent scenario was toward the co-administration of PAZ with CYP3A4 inhibitors, where despite an increased PK AUC_288-312_ ratio being <2.0 (approximately 1.60), based on the PBPK model, it was supported to reduce the clinical dose to a quarter, instead of not to adjust dosage. Another prominent scenario was toward the co-administration of PAZ with acid-reducing agents, where PK AUC_288-312_ reduced by nearly 2-fold, however the simulation of dosing adjustment suggested that there was no need to adjust the dosing regimen, rather than to enhance dose by 2-fold.

##### 3.4.4.4 Key modeling parameters for extremely poor soluble drug in the development of PBPK model

Although there are several studies about the PBPK model of PAZ (Fink et al., 2020a; Fink et al., 2020b; Riedmaier et al., 2020), to our knowledge, this is the first study that implemented a PBPK model to explore dosing adjustment of PAZ. The pH-solubility data is very important to the PBPK development of PAZ owing to its extremely aqueous solubility and pH-dependent property. Hence, pH-solubility is a key parameter for the PBPK model of PAZ. The solubility data reflecting PAZ’s true gastrointestinal solubility (e.g., 1) solubility data at pH = 4.78 in the stomach at steady state after taking esomeprazole; 2) solubility data at pH = 4.9 in the stomach when high-fat food consumption; and 3) gastrointestinal solubility alteration in patients with liver dysfunction) were experimentally determined and loaded into the PBPK model to ensure the predictive performance of the model. Additionally, to assess the DDI, CYP3A4CL_int_ data, a key modeling parameter in the assessment of DDI, was experimentally determined using the substrate depletion method. Furthermore, PPSF is also a key parameter for the simulation of PAZ PK variables in patients with hepatic impairment. A PBPK approach is a well-established tool for the prediction of dosing adjustment of a drug under multiple clinical situations (Zhuang and Lu, 2016). The recommendations for dosing adjustment with the PBPK model have been reported in many papers, involving dosing adjustment in DDIs (Saeheng et al., 2020), with food intake (Riedmaier et al., 2020), hepatic insufficient patients (Han et al., 2021; Heimbach et al., 2021), and for pediatric dose selection (Templeton et al., 2018). In these papers ^[25,^ ^55–57]^, it can be found that the key properties include metabolizing data of CYP enzyme, plasma protein binding, pH-solubility, effect of altered bile salts on solubility in patients with insufficient hepatic function, and altered physiological parameters after food intake, for accurate prediction with the PBPK model. It is certain that the development of the PAZ PBPK model incorporates many key parameters confirmed by the published papers.

## 4 Conclusion

In this study, the PBPK mathematical model of PAZ was developed successfully under multiple clinical situations involving concomitant use with CYP3A4 inhibitors, with acid-reducing agents, with food, and in patients with hepatic impairment. Furthermore, the PBPK model was then used to determine the suitable dosage regimen in the above four situations to maximize the clinical efficacy and minimize the adverse events or therapeutic failure. In summary, the developed PBPK models have successfully predicted the PK variables and profiles of PAZ in various different clinical situations. A dosage adjustment strategy for multiple clinical uses was suggested to use this PBPK model, instead off only considering AUC ratio of PK.

## Data Availability

The original contributions presented in the study are included in the article/Supplementary Material, further inquiries can be directed to the corresponding authors.
