# Supplementary material for: Prediction for optimal dosage of pazopanib under various clinical situations using physiologically based pharmacokinetic modeling
Source: Front Pharmacol. 2022 Sep 12;13:963311. doi: 10.3389/fphar.2022.963311 (PMC9510668; doi:10.3389/fphar.2022.963311)
Supplement: Supplementary file 1 [file DataSheet2.docx]

| **Supplementary Table S1** Input parameters used for ketoconazole in PBPK model |
| --- |

| Property | Values | Source | Descriptions |
| --- | --- | --- | --- |
| MW(g·mol^-1^) | 531.4 | [1] | Molecular weight |
| pKa (base) | 6.51 |  | Base dissociation constant |
| Log (@pH7.4) | 2.67 |  | Lipophilicity |
| Solubility(μg/mL, @pH6.5) | 6.93 |  | Solubility in buffer |
| P_eff_ (🞨10^-5^ cm⋅s^-1^) | 1.24 |  | Human permeability |
| f_up_ | 0.015 |  | Fraction of free drug in plasma |
| Rbp | 0.59 |  | Blood-to-plasma concentration ratio |
| CYP3A4 V_max_ (pmol/min/pmol) | 4.0 | Optimized | Maximum metabolism velocity for CYP3A4 |
| CYP3A4 K_m_ (μM) | 15.0 |  | Michaelis-Menten constant for CYP3A4 |
| UGT1A1 V_max_ (pmol/min/pmol) | 9.37 | [2] | Maximum metabolism velocity for UGT1A1 |
| UGT1A1 K_m_ (μM) | 22.3 |  | Michaelis-Menten constant for UGT1A1 |
| CL_R_(L/h) | 0.11 | — | Renal clearance |
| Partition coefficients | Rodgers and Rowland |  | Calculation method from cell to plasma coefficients |
| Cellular permeabilities | PK-Sim Standard |  | Permeability calculation method across cell |
| Weibull time (min) | 120 | Optimized | Dissolution time of 50% drug |
| Weibull shape | 0.92 |  | Shape parameter of Weibull function |
| K_i_ CYP3A4(μM) | 0.015 | [1] | Inhibition constant on CYP3A4 |
| K_i_ P-gp | 2.27 | [2] | Inhibition constant on P-gp |

| **Supplementary Table S2** Input parameters used for lapatinib in PBPK model |
| --- |

| Property | Values | Source | Descriptions |
| --- | --- | --- | --- |
| MW(g·mol^-1^) | 581.06 | Chemspider | Molecular weight |
| pKa(base) | 4.6, 6.7 | [3] | Base dissociation constant |
| LogP | 4.8 | Optimized | Lipophilicity |
| Water Solubility in FaSSIF pH=6.5(μg/mL) | 5.4 | [3] | Solubility in FaSSIF media |
| P_eff_ (🞨10^-4^ cm⋅s^-1^) | 1.84 |  | Human permeability |
| f_up_ | 0.01 | [4] | Fraction of free drug in plasma |
| Rbp | 0.84 | [5] | Blood-to-plasma concentration ratio |
| CPY3A4 CL_int_ (μL/min/nmol) | 105 | [6] | Intrinsic clearance for CYP3A4 |
| CPY3A5 CL_int_ (μL/min/nmol) | 5 |  | Intrinsic clearance for CYP3A5 |
| Partition coefficients | Rodgers and Rowland | Optimized | Calculation method from cell to plasma coefficients |
| Cellular permeabilities | PK-Sim standard | Optimized | Permeability calculation method across cell |
| K_i_ CPY3A4 (μM) | 2.68 | [7] | Inhibition constant on CPY3A4 |
| k_inact_ CPY3A4 (min^-1^) | 0.021 |  | The maximum rate of inactivation against CYP3A4 |
| K_i_ P-gp (μM) | 3.9 | [8] | Inhibition constant on P-gp |
| K_i_ BCRP1 (μM) | 0.025 |  | Inhibition constant on BCRP1 |
| K_i_ OATP1B1(μM) | 4.0 |  | Inhibition constant on OATP1B1 |

**Supplementary Table S3** Comparison of physiological parameters used in fasted and fed state

| Compartment | Condition | pH^a^ | Transit time (h)^b^ | Volume (mL)^c^ |
| --- | --- | --- | --- | --- |
| Stomach | Fasted | 1.6 | 0.4 | 170 (default) |
|  | Fed (low-fat) | 1.6 | 0.7 | 600 |
|  | Fed (high-fat) | 4.9 | 1.0 | 1000.0 |
| Duodenum | Fasted | 6.0 |  |  |
|  | Fed (low-fat) | 6.0 |  |  |
|  | Fed (high-fat) | 5.4 |  |  |
| Upper jejunum | Fasted | 6.2 |  |  |
|  | Fed (low-fat) | 6.2 |  |  |
|  | Fed (high-fat) | 5.4 |  |  |
| Lower jejunum | Fasted | 6.4 |  |  |
|  | Fed (low-fat) | 6.4 |  |  |
|  | Fed (high-fat) | 6.0 |  |  |
| Upper ileum | Fasted | 7.2 |  |  |
|  | Fed (low-fat) | 7.2 |  |  |
|  | Fed (high-fat) | 7.2 |  |  |
| Lower ileum | Fasted | 7.2 |  |  |
|  | Fed (low-fat) | 7.2 |  |  |
|  | Fed (high-fat) | 7.2 |  |  |
| Cecum | Fasted | 6.4 |  |  |
|  | Fed (low-fat) | 6.8 |  |  |
|  | Fed (high-fat) | 6.6 |  |  |
| Colon | Fasted | 6.4 |  |  |
|  | Fed (low-fat) | 6.8 |  |  |
|  | Fed (high-fat) | 6.6 |  |  |
| Rectum | Fasted | 6.4 |  |  |
|  | Fed (low-fat) | 6.8 |  |  |
|  | Fed (high-fat) | 6.6 |  |  |

^a^: Gastrointestinal pH. ^b^: Gastric transit time before meal and after meal.

^c^: Volume of stomach before meal and after meal

**Supplementary Table S4** Fractional changes of physiological parameter in patients with impaired hepatic function in the PBPK-BO model with respect to healthy individuals

| Physiological parameters | Impaired patients by stage | | | Source |
| --- | --- | --- | --- | --- |
|  | Mild ^a^ | Moderate ^b^ | Severe ^c^ |  |
| Hematocrit Value | 0.91 | 0.86 | 0.81 | [43] |
| Blood flow |  |  |  |  |
| renal | 0.88 | 0.65 | 0.48 |  |
| hepatic arterial | 1.4 | 1.6 | 1.9 | [44] |
| Liver volume fraction | 0.89 | 0.71 | 0.61 | [44] |
| Albumin | 0.8 | 0.7 | 0.6 |  |
| GFR | 1 | 0.7 | 0.36 | [43] |
| Gastric residence time | 1.2 | 1.4 | 1.5 |  |
| Small intestinal transit time | 1.2 | 1.38 | 1.5 | [44] |
| Colon transit time | 2 | 2 | 2 |  |
| CYP3A4 activity | 0.79 | 0.57 | 0.23 |  |
| PPSF | 1.25 | 1.43 | 1.67 | Calculated |
| Gastrointestinal solubility | 0.8 | 0.5 | 0.2 |  |

^a,b,c^: Calculated by dividing mean of normal subjects with mild (a), moderate (b) and severe (c), respectively.

**Table S5** Effect of NaTC on the PAZ solubility

| NaTC (mM) | Solubility (μg/mL) at Fasted state | SD |
| --- | --- | --- |
| 0 | 0.36 | 0.06 |
| 0.937 | 0.27 | 0.06 |
| 1.87 | 0.40 | 0.07 |
| 3.75 | 1.51 | 0.35 |
| 7.5 | 3.02 | 0.41 |
| 15 | 6.93 | 0.78 |
| 30 | 27.9 | 2.3 |

**Table S6** Calculated in vitro intrinsic clearance metabolized by CYP3A4

| Enzyme | K (min^-1^)^a^ | V (ml)^b^ | Enzyme concentraion^c^ | CL_int_ (μl/min/pmol)^d^ |
| --- | --- | --- | --- | --- |
| CYP3A4 | 0.0053 | 1.0 | 5 pmol | 1.10 |

^a^: K is rate constant metabolized by CYP3A4 and calculated using equation 1

^b^: V is the final reaction volume of *in vitro* metabolism experiments

^c^: CYP3A4 concentration

^d^: Calculated using equation 2

**Supplementary Table S7** Comparisons of mean PK variables (arithmetic mean) between model predictions and clinical observations in humans following multiple dosing regimen

| Clinical study | PK variables | | Prediction | Observation | Prediction/observation ratio |
| --- | --- | --- | --- | --- | --- |
| 50 mg OD for 21 consecutive days | Day 1 | C_max_ (μg·mL^-1^) | 4.7 (2.9-7.2) | 3.3 | 1.42 |
|  |  | C_trough_ (μg·mL^-1^) | 2.2 (1.2-3.0) | 1.5 | 1.47 |
|  |  | AUC_0-24_ (μg·h·mL^-1^) | 63.1 (37.4-85.1) | 51.8 | 1.22 |
|  |  | T_max_(h) | 2.3 (1.1-3.0) | 3.0 | 0.77 |
|  | Day 22 | C_max_ (μg·mL^-1^) | 9.0 (5.5-12.9) | 8.4 | 1.07 |
|  |  | C_trough_ (μg·mL^-1^) | 4.8 (2.7-4.0) | 5.1 | 0.94 |
|  |  | AUC_504-528_ (μg·h·mL^-1^) | 112.1 (66.5-151.3) | 157.7 | 0.71 |
|  |  | T_max_(h) | 1.5 (1.3-1.8) | 3.0 | 0.50 |
| 100 mg OD for 21 consecutive days | Day 1 | C_max_ (μg·mL^-1^) | 7.8 (5.4-11.0) | 6.5 | 1.20 |
|  |  | C _trough_ (μg·mL^-1^) | 3.7 (2.4-5.0) | 2.8 | 1.32 |
|  |  | AUC_0-24_ (μg·h·mL^-1^) | 119.1 (77.6-158.2) | 96.9 | 1.23 |
|  |  | T_max_(h) | 2.5 (1.3-3.5) | 4.0 | 0.63 |
|  | Day 22 | C_max_ (μg·mL^-1^) | 13.9 (10.3-20.2) | 11.0 | 1.26 |
|  |  | C_trough_ (μg·mL^-1^) | 7.6 (4.6-9.8) | 4.9 | 1.55 |
|  |  | AUC_504-528_ (μg·h·mL^-1^) | 208.4 (135.7-276.9) | 160.7 | 1.30 |
|  |  | T_max_(h) | 1.5 (1.3-1.8) | 8.0 | 0.19 |
| 200 mg OD for 21 consecutive days | Day 1 | C_max_ (μg·mL^-1^) | 11.8 (9.3-14.8) | 7.5 | 1.57 |
|  |  | C _trough_ (μg·mL^-1^) | 5.3 (4.3-7.0) | 2.9 | 1.83 |
|  |  | AUC_0-24_ (μg·h·mL^-1^) | 171.7 (118.2-271.5) | 104.5 | 1.64 |
|  |  | T_max_(h) | 2.3 (1.2-3.0) | 3.0 | 0.77 |
|  | Day 22 | C_max_ (μg·mL^-1^) | 23.2(17.8-28.3) | 23.7 | 0.98 |
|  |  | C_trough_ (μg·mL^-1^) | 11.6 (6.1-18.6) | 12.4 | 0.94 |
|  |  | AUC_504-528_ (μg·h·mL^-1^) | 360.6 (248.3-4546.8) | 384.8 | 0.94 |
|  |  | T_max_(h) | 2.0 (1.6-2.8) | 2.0 | 1.00 |
| 300 mg twice daily for 21 consecutive days | Day 1 | C_max_ (μg·mL^-1^) | 14.1 (11.9-17.3) | 8.4 | 1.68 |
|  |  | C_trough_ (μg·mL^-1^) | 10.1(8.5-12.6) | 4.1 | 2.46 |
|  |  | AUC_0-24_ (μg·h·mL^-1^) | 173.4 (114.0-218.6) | 120.1 | 1.44 |
|  |  | T_max_(h) | 1.9 (1.7-3.3) | 4.0 | 0.48 |
|  | Day 22 | C_max_ (μg·mL^-1^) | 43.0 (33.4-56.0) | 36.7 | 1.17 |
|  |  | C_trough_ (μg·mL^-1^) | 34.3 (18.7-48.5) | 29.5 | 1.16 |
|  |  | AUC_504-528_ (μg·h·mL^-1^) | NC | NC | - |
|  |  | T_max_(h) | 1.5 (1.3-2.0) | 4.0 | 0.38 |
| 400 mg OD for 21 consecutive days | Day 1 | C_max_ (μg·mL^-1^) | 15.7 (13.6-18.9) | 10.3 | 1.52 |
|  |  | C_trough_ (μg·mL^-1^) | 7.6 (5.3-9.0) | 3.9 | 1.95 |
|  |  | AUC_0-24_ (μg·h·mL^-1^) | 225.3 (147.3-295.7) | 142.8 | 1.58 |
|  |  | T_max_(h) | 2.5 (1.9-3.5) | 4.0 | 0.63 |
|  | Day 22 | C_max_ (μg·mL^-1^) | 29.8 (25.2-36.1) | 21.8 | 1.37 |
|  |  | C_trough_ (μg·mL^-1^) | 15.1 (7.3-26.6) | 10.4 | 1.45 |
|  |  | AUC_504-528_ (μg·h·mL^-1^) | 500.7 (327.4-657.2) | 447.1 | 1.12 |
|  |  | T_max_(h) | 1.8(1.5-2.5) | 3.0 | 0.60 |
| 400 mg twice daily for 21 consecutive days | Day 1 | C_max_ (μg·mL^-1^) | 14.5 (13.6-18.9) | 6.5 | 2.23 |
|  |  | C _trough_ (μg·mL^-1^) | 11.0 (9.7-13.8) | 3.3 | 3.33 |
|  |  | AUC_0-24_ (μg·h·mL^-1^) | 321.8 (211.5-424.3) | 365.4 | 0.88 |
|  |  | T_max_(h) | 2.3 (1.9-3.5) | 3.0 | 0.77 |
|  | Day 22 | C_max_ (μg·mL^-1^) | 52.7 (37.7-67.1) | 36.1 | 1.46 |
|  |  | C _trough_ (μg·mL^-1^) | 38.7 (21.1-58.3) | 28.1 | 1.38 |
|  |  | AUC_504-528_ (μg·h·mL^-1^) | NC | NC | - |
|  |  | T_max_(h) | 1.8 (1.2-2.5) | 3.1 | 0.58 |
| 600 mg OD for 21 consecutive days | Day 1 | C_max_ (μg·mL^-1^) | 18.0 (15.7-21.2) | 11.2 | 1.61 |
|  |  | C _trough_ (μg·mL^-1^) | 8.8 (5.9-10.3) | 4.7 | 1.87 |
|  |  | AUC_0-24_ (μg·h·mL^-1^) | 290.8 (188.3-400.4) | 157.6 | 1.85 |
|  |  | T_max_(h) | 2.8 (2.3-3.8) | 3.0 | 0.93 |
|  | Day 22 | C_max_ (μg·mL^-1^) | 34.3 (28.8-44.2) | 18.2 | 1.88 |
|  |  | C_trough_ (μg·mL^-1^) | 16.1 (8.1-32.6) | 11.0 | 1.46 |
|  |  | AUC_504-528_ (μg·h·mL^-1^) | 581.5 (376.5-800.3) | 333.7 | 1.74 |
|  |  | T_max_(h) | 2.0 (1.5-3.0) | 6.0 | 0.33 |
| 800 mg OD for 21 consecutive days | Day 1 | C_max_ (μg·mL^-1^) | 20.4 (17.1-23.1) | 19.4 | 1.05 |
|  |  | C_trough_ (μg·mL^-1^) | 9.7 (6.3-11.2) | 9.4 | 1.03 |
|  |  | AUC_0-24_ (μg·h·mL^-1^) | 317.4 (204.1-446.4) | 275.1 | 1.15 |
|  |  | T_max_(h) | 2.5 (2.3-4.0) | 3.5 | 0.71 |
|  | Day 22 | C_max_ (μg·mL^-1^) | 39.5 (31.1-49.6) | 45.1 | 0.88 |
|  |  | C _trough_ (μg·mL^-1^) | 20.6 (8.8-36.7) | 24.0 | 0.86 |
|  |  | AUC_504-528_ (μg·h·mL^-1^) | 666.5 (428.5-937.3) | 743.3 | 0.90 |
|  |  | T_max_(h) | 2.8 (2.3-3.5) | 2.0 | 1.40 |
| 1000 mg OD for 21 consecutive days | Day 1 | C_max_ (μg·mL^-1^) | 21.6 (18.4-24.7) | 36.1 | 0.60 |
|  |  | C_trough_ (μg·mL^-1^) | 10.4 (6.6-2.1) | 12.2 | 0.85 |
|  |  | AUC_0-24_ (μg·h·mL^-1^) | 337.4 (215.4-478.5) | 400.0 | 0.84 |
|  |  | T_max_(h) | 2.8 (2.0-4.0) | 2.0 | 1.40 |
|  | Day 22 | C_max_ (μg·mL^-1^) | 42.0 (32.8-53.4) | 53.2 | 0.79 |
|  |  | C_trough_ (μg·mL^-1^) | 22.0 (9.4-39.5) | 28.8 | 0.76 |
|  |  | AUC_504-528_ (μg·h·mL^-1^) | 708.4 (452.3-1004.7) | 796.1 | 0.89 |
|  |  | T_max_(h) | 2.3 (2.0-3.5) | 4.5 | 0.51 |
| 1400 mg OD for 21 consecutive days | Day 1 | C_max_ (μg·mL^-1^) | 22.7 (20.0-27.3) | 20.8 | 1.09 |
|  |  | C_trough_ (μg·mL^-1^) | 11.5 (7.2-13.5) | 8.2 | 1.40 |
|  |  | AUC_0-24_ (μg·h·mL^-1^) | 367.4 (231.4-521.8) | 291.6 | 1.26 |
|  |  | T_max_(h) | 3.0 (2.0-4.0) | 3.0 | 1.00 |
|  | Day 22 | C_max_ (μg·mL^-1^) | 43.1(35.5-58.8) | 32.7 | 1.32 |
|  |  | C_trough_ (μg·mL^-1^) | 20.4 (10.3-43.7) | 16.6 | 1.23 |
|  |  | AUC_504-528_ (μg·h·mL^-1^) | 771.5 (485.9-1095.7) | 536.2 | 1.44 |
|  |  | T_max_(h) | 2.8 (2.3-3.6) | 2.0 | 1.40 |
| 2000 mg OD for 21 consecutive days | Day 1 | C_max_ (μg·mL^-1^) | 25.1 (21.5-30.8) | 44.5 | 0.56 |
|  |  | C_trough_ (μg·mL^-1^) | 12.8 (7.9-15.3) | 12.2 | 1.05 |
|  |  | AUC_0-24_ (μg·h·mL^-1^) | 480.5 (296.6-675.9) | 681.2 | 0.71 |
|  |  | T_max_(h) | 2.8 (2.3-4.3) | 4.0 | 0.70 |
|  | Day 22 | C_max_ (μg·mL^-1^) | 50.9 (38.3-64.4) | 52.7 | 0.97 |
|  |  | C_trough_ (μg·mL^-1^) | 27.0 (11.2-47.9) | 28.8 | 0.94 |
|  |  | AUC_504-528_ (μg·h·mL^-1^) | 840.8 (519.0-1182.8) | 860.3 | 0.98 |
|  |  | T_max_(h) | 3.0 (2.5-4.0) | 3.0 | 1.00 |

**Supplementary Table S8** The mean observed and predicted PK variables for the CYP3A4 inhibitors according to PBPK model

| Drug | Variables | Predicted | Observed | Predicted/Observed |
| --- | --- | --- | --- | --- |
| Ketoconazole | C_max_ (μg·mL^-1^) | 6.0 | 6.2 | 0.97 |
|  | AUC_0-48_ (μg·h·mL^-1^) | 39.98 | 40.6 | 0.98 |
|  | T_max_ | 1.6 | 1.5 | 1.07 |
|  | C_max_ (ng·mL^-1^) | 362.5 | 311.0 | 1.17 |
| Lapatinib | AUC_0-48_ (ng·h·mL^-1^) | 6123.5 | 5328.9 | 1.15 |
|  | T_max_ | 2.0 | 3.0 | 0.67 |
