# Supplementary material for: Prediction for optimal dosage of pazopanib under various clinical situations using physiologically based pharmacokinetic modeling
Source: Front Pharmacol. 2022 Sep 12;13:963311. doi: 10.3389/fphar.2022.963311 (PMC9510668; doi:10.3389/fphar.2022.963311)
Supplement: Supplementary file 2 [file DataSheet1.docx]

**Figure S1 PAZ depletion by CYP3A4.**

The red squares () refer to the remaining PAZ (%) at different time points. The data shown were obtained from a single experiment conducted in triplicate. Each data point indicates the mean ± S.D. (n = 3).

**

**

**Figure S2 The mean predicted and observed plasma concentration-time profiles of ketoconazole (A) and lapatinib (B) in healthy human.** The red (🞏) and blue (🞏) squares refer to clinical observed pharmacokinetic data of ketoconazole and lapatinib, respectively.
